# Zebrafish as a translational platform for investigating multi-organ pharmacological interactions of traditional Chinese medicine

**DOI:** 10.3389/fphar.2026.1785275

**Published:** 2026-06-26

**Authors:** Jianhua Guo, Jinmin Ma, Guobao Li, Yan Pi

**Affiliations:** 1 Key Lab of Artificial Organs and Computational Medicine, Institute of Translational Medicine, Shulan International Medical College, Zhejiang Shuren University, Hangzhou, Zhejiang, China; 2 Medical Frontier Innovation Research Center, The First Hospital of Lanzhou University, Lanzhou, China; 3 School of Resources and Environment, Baoshan University, Baoshan, Yunnan, China; 4 State Key Laboratory of Genetic Engineering, National Demonstration Center for Experimental Biology Education, School of Life Sciences, Fudan University, Shanghai, China

**Keywords:** multi-organ platform, precision delivery, research paradigm, traditional Chinese medicine, zebrafish model

## Abstract

The advancement of Traditional Chinese Medicine (TCM) requires model systems capable of dissecting its multi-component, multi-target pharmacology in a rapid and systematic manner. This review presents the zebrafish as a versatile *in vivo* pharmacological platform for modern TCM research, not merely a screening tool. We synthesize its recent applications across cardiovascular, neuropsychiatric, metabolic, and oncological diseases, emphasizing how optical transparency and genetic tractability enable real-time quantification of multi-target interactions - for instance, dose-dependent heart rate changes, neutrophil migration distances, and tumor fluorescence intensities - thereby linking molecular pathways to whole-organism phenotypic outcomes. A dedicated section critically evaluates its role in mechanistic safety assessment, moving beyond descriptive toxicology toward elucidation of adverse outcome pathways. We then identify current limitations where the zebrafish’s potential is underutilized, such as in resolving the spatiotemporal dynamics of herbal formula compatibility. Finally,we propose “zebrafish-plus” paradigms integrating organoids, single-cell multi-omics, and AI-driven phenotypic analytics. These frameworks are designed to generate testable mechanistic hypotheses and perform early efficacy/safety profiling, while explicitly recognizing that all zebrafish-derived findings require rigorous validation in mammalian models before clinical interpretation.

## Graphical Abstract

**Figure:**
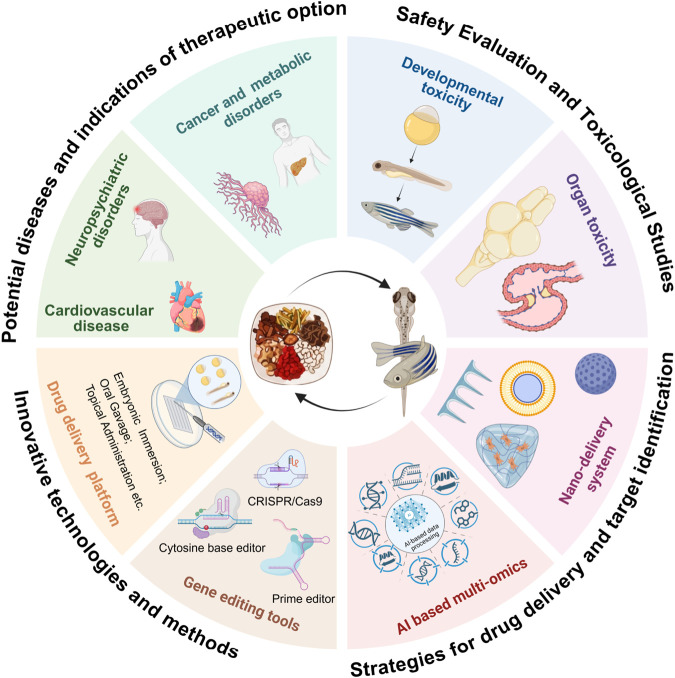

## 1 Introduction

As an integral component of TCM, herbal medicine has demonstrated distinctive advantages in recent years for disease treatment and health maintenance, particularly in personalized therapeutic regimens and the practice of the “preventive treatment of disease” philosophy. Research utilizing animal models based on TCM theory serves as a critical tool for elucidating TCM principles, developing new formulations, and evaluating the efficacy and mechanisms of herbal interventions (Wang et al., 2021a). Nevertheless, this field faces several significant challenges. The lack of well-defined molecular biomarkers for TCM syndromes compromises the reproducibility of animal models (Lin et al., 2025); A persistent gap exists between animal models and clinical populations, as most studies employ young animals to simulate age-related diseases, overlooking crucial factors such as age, sex, and constitutional compatibility (Ma et al., 2024); Current models often prioritize symptom mimicry over the integration of TCM pathogenesis theory, thereby weakening their clinical relevance (Wang S. et al., 2025; Eng et al., 2019); Furthermore, existing evaluation systems rely predominantly on Western medical indicators, while TCM diagnostic parameters lack quantifiable metrics and standardized scoring criteria (Jia et al., 2021; Yao et al., 2022; Zhou X. et al., 2019); Environmental variables may affect model stability, and inadequate experimental durations frequently undermine the reliability of results (Zhou X. et al., 2019). These limitations underscore the urgent need to develop animal models that authentically embody TCM theoretical principles while incorporating modern scientific validation frameworks. These limitations highlight the urgent need to develop animal models that authentically reflect TCM principles while incorporating modern scientific validation frameworks.

Zebrafish (*Danio rerio*) has become a prominent model organism, particularly valuable for bridging holistic traditional medicine with modern molecular inquiry. Its key advantages include high genomic conservation with humans (∼87%), optical transparency of embryos enabling real-time *in vivo* observation, a short reproductive cycle (3–4 months to sexual maturity), and cost-effective maintenance (Bedell et al., 2025; Srivastava et al., 2025). These traits make zebrafish ideal for developmental biology, disease modeling, and pharmaceutical screening. The external development of embryos facilitates high-throughput *in vivo* assays, such as 96-well plate-based drug screening (Macrae and Peterson, 2023). CRISPR/Cas9-mediated genome editing allows efficient generation of human disease models, including those for Parkinson’s disease and cancer (Bedell et al., 2025; Srivastava et al., 2025; Wang PC. et al., 2024). With both innate and adaptive immune systems, zebrafish is widely used in toxicology, pharmacology, and neuroscience research (Srivastava et al., 2025; Okitsu-Sakurayama et al., 2025; Vaz-Rodrigues and De La Fuente, 2025). Additionally, embryos under 5 days post-fertilization are exempt from certain animal ethics regulations, streamlining protocols and enhancing its role as a translational platform (Bedell et al., 2025; Srivastava et al., 2025).

In TCM research, zebrafish has demonstrated unique value in pharmacological efficacy evaluation, toxicity mechanism studies, and metabolic investigation (Macrae and Peterson, 2023). For efficacy assessment, it enables screening of herbal active components and evaluation of compound formulations. For example, in infection and immunodeficiency models, Shenfu Huang Formula was shown to reduce neutrophil infiltration, enhance macrophage function, and attenuate thrombosis by inhibiting NF-κB, offering a multi-target strategy for COVID-19 complications (Liu et al., 2020). In toxicity studies, zebrafish accurately reflects TCM toxicity profiles, aiding identification of toxic components, target organs, and pathways. Examples include Tripterygium wilfordii-induced cardiotoxicity (Sun et al., 2025) and Euphorbia kansui-related hepatotoxicity (Zhao et al., 2019), the latter linked to altered expression of detoxification genes (e.g., CYP3A4, UGT1A1) and oxidative stress. Metabolically, zebrafish shares >70% homology with mammals in metabolic enzymes, enabling precise mapping of herbal compound metabolism, as illustrated by studies on calycosin Phase I/II conversions and transport (Hu et al., 2012; Lu X. et al., 2021). These capabilities establish zebrafish as an indispensable tool for TCM modernization, supporting high-throughput screening, cost-effective testing, and ethical compliance, thereby advancing evidence-based internationalization of Chinese medicine.

Consequently, there remains an urgent need for a model organism capable of capturing the systemic nature of TCM while allowing granular molecular dissection. We argue that the zebrafish model uniquely fulfills this dual requirement. Its role extends beyond efficient screening; it serves as a living biosensor and a dynamic integrator for the complex bioactivity of TCM. This review will reinterpret recent progress through this lens, critically assess the model’s transformative role in safety evaluation, and ultimately outline a course for leveraging zebrafish not merely to validate TCM, but to fundamentally decode the principles underlying its holistic efficacy.

Therefore, this review examines the zebrafish as a tractable pharmacological platform for systematically deconstructing the multi-target mechanisms of TCM interventions. By synthesizing evidence across major disease areas and safety assessment, we highlight how real-time imaging and genetic tools enable quantitative, dose response-based dissection of herbal drug actions - from single-compound pharmacokinetics to multi-herb network effects - while acknowledging the model’s physiological differences from mammals. We further outline “zebrafish-plus” paradigms that aim to enhance translational relevance, shifting the focus from descriptive validation to mechanism-oriented inquiry that can inform subsequent mammalian studies.

## 2 Research progress of TCM based on zebrafish models: from phenotypic observation to mechanism exploration

### 2.1 Cardiovascular disease

The zebrafish model offers unparalleled advantages for cardiovascular research, primarily due to its optical transparency, genetic tractability, and conserved cardiovascular physiology. These features enable real-time *in vivo* visualization of heart development, vascular remodeling, thrombosis, and lipid metabolism—making it an ideal system for dissecting the multi-target mechanisms of TCM (Table 1).

**TABLE 1:** Zebrafish as an integrative model for TCM in cardiovascular research.

Research focus	Representative TCM interventions	Major targets/Pathways	Integrated pharmacological effects	Model limitations
Myocardial protection	Zhenwu Decoction, Qiangxinyin, Astragalus polysaccharide, ginseng glycopeptide APMCG-1	sGC-cGMP-PKG pathway, calcium influx, mitochondrial function, Bax/Bcl-2, Caspase-3/9, PI3K/AKT.	Integrated network modulating calcium homeostasis, mitochondrial function, apoptosis, inflammation, and oxidative stress; alleviates hypertrophy, fibrosis, and heart failure	1. Anatomical, metabolic, and chronic pathological differences from humans 2. Focus on isolated pathways; lack of systematic analysis of spatiotemporal crosstalk among cell types (endothelial, cardiomyocyte, immune) and remodeling of the cardiovascular metabolic-immune microenvironment
Angiogenesis modulation	Compound Danshen Dripping Pills (pro-angiogenic), Notoginsenoside R1 (pro-angiogenic), Timosaponin AIII (anti-angiogenic), Polygonum cuspidatum extract (anti-angiogenic)	VEGFA/Kdrl, PI3K/Akt, Ang2-Tie2 axis, VEGFR2, Akt/ERK/eNOS.	Context-dependent bidirectional regulation centered on VEGF: promotes ischemic repair or inhibits pathological angiogenesis	
Antithrombotic activity	Xanthotoxin, Alnustone, earthworm protein EPF3/DPf3, Danshen-Chuanxiong herb pair, Leonurine, Geniposide and citric acid, Salvianolic acid B, Tetramethylpyrazine	IL-1R1-MEK/ERK, IL-17A/IL-17RA/Src/RAC1/MEK/ERK, NF-κB, PI3K-Akt, Rap1, coagulation factor F11	Bidirectional regulation of hemostasis: promotes platelet production or exerts antithrombotic effects via multi-target synergy (coagulation factors, platelet aggregation, antioxidant, anti-inflammatory)	
Anti-atherosclerosis and lipid metabolism	Oleanolic Acid nanoparticles, Dendrobium huoshanense polysaccharide, Typhae pollen polysaccharide TPP-4, Acanthopanax senticosus	JNK/MAPK, CYP7A1, eNOS/VEGFA, ET-1	Multi-pathway synergy: regulates lipid metabolism, suppresses inflammation, reduces oxidative stress, and improves endothelial function	
Cardiovascular repair and regeneration	Mongolian medicine Erdun-Wurile, Buxue Yimu Pills, Panax notoginseng flower saponins, danshensu derivative ADTM.	MVDA, VEGFR2, VEGF, L-type calcium channels	Coordinates angiogenesis, cell proliferation/apoptosis, and extracellular matrix remodeling to drive tissue healing and functional recovery	

#### 2.1.1 Myocardial protection

**FIGURE 1:**
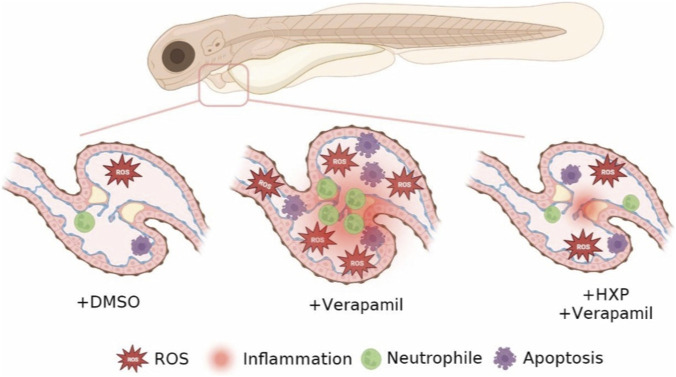
Diagram illustrating the mechanism by which Huoxin pill (HXP) alleviates verapamil-induced heart failure (HF) in zebrafish embryos (Li et al., 2024a). Verapamil disrupts cardiomyocyte calcium homeostasis, triggering a pathological cascade of ROS generation, local inflammation, neutrophil infiltration, and cardiomyocyte apoptosis. This oxidative stress–inflammation–apoptosis axis collectively precipitates contractile dysfunction and heart failure. Co-administration of HXP attenuates these pathological changes, suggesting multi-target cardioprotective effects involving antioxidant, anti-inflammatory, and anti-apoptotic activities. Reproduced with permission (Li et al., 2024a). Copyright © 2024, Elsevier.

Zebrafish models allow high-throughput analysis of the multi-component, multi-target cardioprotective effects of TCM. By linking molecular perturbations to phenotypic readouts such as heart rate, pericardial oedema, and cardiac output, they help to define mechanism–phenotype relationships (Figure 1).

Several studies have shown that TCM compounds engage multiple pathways. For instance, Zhenwu Decoction alleviates myocardial hypertrophy and fibrosis by activating the sGC-cGMP-PKG pathway (Chen L. et al., 2023), while Qiangxinyin counters isoproterenol-induced cardiac hypertrophy by blocking calcium influx and improving mitochondrial function, with psoralen, kaempferol, and icaritin as likely active constituents (Zhou et al., 2024). However, it remains unclear whether the cGMP-PKG axis and calcium handling operate independently or synergise in the same cellular context. At the monomer level, Astragalus polysaccharide inhibits apoptosis in zebrafish heart failure models by modulating Bax/Bcl-2 and Caspase-3/9, thereby increasing heart rate and ATP levels (Zhou C. et al., 2025), while Ginseng residue glycopeptide APMCG-1 improves cardiac function in diabetic zebrafish through PI3K/AKT activation (Li Z. et al., 2025). Although these studies point to converging effects on apoptosis, oxidative stress, and energy metabolism, the model systems (genetic vs. chemically induced heart failure, diabetic vs. non-diabetic backgrounds) differ substantially, which complicates direct comparison. A systematic dissection of how these pathways intersect across different cardiac cell types and disease stages is still lacking.

#### 2.1.2 Bidirectional regulation of angiogenesis

The transparent vasculature of zebrafish makes it possible to directly observe the formation or regression of intersegmental vessels and to link these vascular phenotypes to signalling events. This has allowed several groups to characterize both pro- and anti-angiogenic activities of TCM components that converge on the VEGF axis.

For example, Compound Danshen Dripping Pills upregulate VEGFA/Kdrl and activate the PI3K/Akt (Hu et al., 2022), whereas Timosaponin AIII suppresses VEGF-induced endothelial migration and impairs ISV development (Zhou et al., 2020). Notoginsenoside R1 promotes angiogenesis via the Ang2-Tie2 axis (Zhong et al., 2020), while Polygonum cuspidatum extract inhibits VEGFR2 and its downstream Akt/ERK/eNOS pathways (Hu et al., 2018) These opposing outcomes likely depend on cellular context, compound concentration, and the specific pathological model employed, but few studies have systematically varied these parameters to define the conditions that dictate the direction of regulation. Angelica sinensis polysaccharide APS2’s pro-angiogenic effect via PI3K/AKT (Zhou MJ. et al., 2025) further illustrates that polysaccharides and small molecules may trigger the same pathway to produce opposite phenotypes, a discrepancy that warrants investigation into receptor-level selectivity and downstream signaling kinetics.

Despite this evidence for bidirectional regulation, the factors that determine whether a given compound acts in a pro- or anti-angiogenic direction within a specific tissue context remain largely unexplored. Systematic comparisons using identical *in vivo* readouts would strengthen the mechanistic framework.

#### 2.1.3 Anti-thrombotic activity

The transparent circulatory system of zebrafish enables simultaneous observation of thrombopoiesis, thrombus formation, and dissolution, making it possible to assess how TCM compounds modulate hemostasis across multiple nodes.

The ability of TCM components to promote platelet production or inhibit thrombosis appears to be highly context-dependent, yet head-to-head comparisons of potency and mechanism under the same experimental conditions are rare. For pro-hematopoietic effects, Xanthotoxin acts through IL-1R1-MEK/ERK (Lai et al., 2023), and Alnustone through the IL-17A/IL-17RA/Src/RAC1/MEK/ERK signaling axis (Li Y. et al., 2024), both ultimately converging on the MEK/ERK module, raising the question of whether they share overlapping downstream effectors or produce synergistic thrombopoietic activity. In contrast, antithrombotic mechanisms are more diverse. Leonurine targets ROS, platelet aggregation, and coagulation factors simultaneously (Liao et al., 2021), Salvianolic acid B binds coagulation factor F11 (Tang et al., 2021), Tetramethyl-pyrazine downregulates multiple coagulation factors (Zhang et al., 2022a), and the herb pair Dan-shen-Chuanxiong acts through synergistic anticoagulation (Li et al., 2020a). Whether these multi-target actions represent true *in vivo* synergy or simply parallel independent activities has not been formally demonstrated. The same limitation applies to formulations such as Gardenia jasminoides extracts, where geniposide and citric acid co-contribute to PI3K-Akt and Rap1 pathway modulation (Shi et al., 2020). These findings point to coordinated control of thrombopoiesis, coagulation factor activity, fibrinolysis, autophagy, and accompanying antioxidant and anti-inflammatory effects. Nonetheless, current studies rarely examine whether modulation of these parallel processes is synergistic, additive, or redundant, which limits the ability to discriminate between pharmacologically meaningful interactions and co-occurring but independent events.

#### 2.1.4 Anti-atherosclerotic effect with modulation of lipid metabolism

Diet-induced or genetic hyperlipidemia models in zebrafish allow non-invasive, longitudinal monitoring of lipid deposition, plaque formation, and endothelial integrity. Reported anti-atherosclerotic effects involve overlapping sets of mechanisms - lipid lowering, anti-inflammation, and endothelial protection, but studies rarely disentangle primary effects on lipid metabolism from secondary benefits conferred by reduced inflammation. For instance, Oleanolic Acid nanoparticles reduce lipid deposition and suppress the JNK/MAPK signaling (Gao et al., 2025), and Dendrobium huoshanense polysaccharide concurrently attenuates plaque formation and neutrophil aggregation (Fan et al., 2020). Typhae pollen polysaccharide TPP-4 reduces vascular permeability and preserves endothelial integrity (Gao et al., 2024), and Acanthopanax senticosus upregulates eNOS/VEGFA while downregulating ET-1 to improve endothelial function (Tian et al., 2025).

The co-occurrence of lipid-regulating and anti-inflammatory properties across structurally diverse compounds suggests either that these activities are intrinsically coupled or that the assays used do not resolve the causal sequence. CYP7A1 upregulation has been proposed as a node linking cholesterol efflux to vascular protection (Jia et al., 2025), but direct evidence that TCM-mediated CYP7A1 induction is necessary for the observed vascular improvements is generally absent.

#### 2.1.5 Cardiovascular repair and regeneration

**FIGURE 2:**
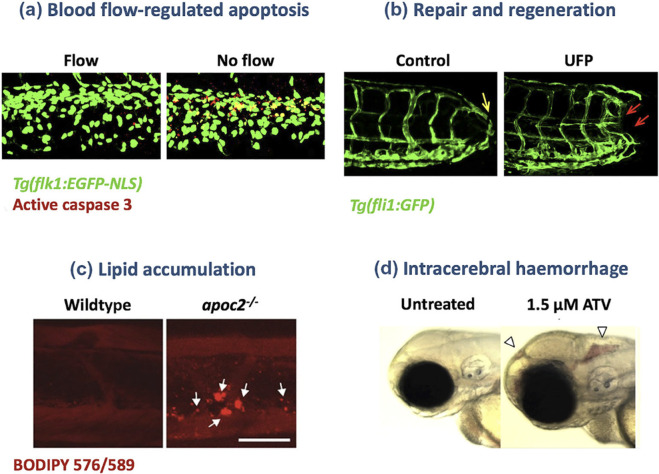
Zebrafish serves as a valuable model for research on vascular repair, regeneration, and lipid accumulation (Bowley et al., 2022). **(a)** Blood flow-regulated apoptosis. Fluorescence labeling (green for blood vessels, red for active Caspase-3 apoptotic cells) visually demonstrates the difference in cell apoptosis under flow versus no flow conditions. This is suitable for studying hemodynamics and cell fate decisions. **(b)** Repair and Regeneration. This compares the regenerative capacity of blood vessels or tissues (green fluorescence) after injury between a control group and a treated group (UFP), applicable for screening compounds that promote repair or regeneration. **(c)** Lipid Accumulation. This compares blood vessels (green) and lipid deposits (red) between wild-type zebrafish and apolipoprotein gene knockout mutants (apoc2^−/−^), serving as a classic model for studying lipid metabolism disorders and related diseases. **(d)** Intracerebral Hemorrhage. This shows the condition of intracranial hemorrhage (red fluorescence) in untreated versus drug-treated (1.5 μM ATV) zebrafish, applicable for evaluating the protective or reparative effects of compounds on cerebrovascular integrity. Reproduced with permission (Bowley et al., 2022). Copyright © 2021, John Wiley and Sons.

Zebrafish are capable of robust cardiac and vascular regeneration, allowing real-time observation of cardiomyocyte proliferation and vessel regrowth (Figure 2). Multiple TCM interventions have been shown to promote cardiac and vascular repair, but the regenerative outcomes are difficult to compare because studies differ in injury models, time points, and endpoints. For instance, the Mongolian medicine Erdun-Wurile enhances cardiomyocyte proliferation and reducing apoptosis via MVDA (Chen X. et al., 2025), Buxue Yimu Pills upregulate VEGFR2 to improve vascular repair (Zhang LL. et al., 2022), and Panax notoginseng flower saponins promote angiogenesis in the infarct border zone through VEGF upregulation (Yang et al., 2016). Furthermore, danshensu derivative ADTM supports repair by blocking L-type calcium channels and stimulating VEGF secretion (Cui et al., 2018). While these reports collectively suggest that coordinated regulation of angiogenesis, apoptosis, and matrix remodeling supports repair, it remains unclear whether the observed tissue restoration is driven mainly by direct effects on proliferating cells or by paracrine signaling from surrounding cell types. Standardized regenerative endpoints across studies would greatly improve cross-study interpretability.

The zebrafish model can link molecular mechanisms to cardiovascular phenotypes with a level of temporal and spatial resolution that is difficult to achieve in rodents. Nevertheless, the existing literature is dominated by single-pathway analyses. Hardly any study has systematically addressed how TCM affects the interplay between endothelial cells, cardiomyocytes, and immune cells, or how it remodels the cardiovascular metabolic–immune microenvironment. Moreover, differences between zebrafish and humans in cardiovascular anatomy (single-chambered heart), drug metabolism, and the time course of chronic pathologies such as atherosclerosis mean that acute observations in embryos or larvae cannot be directly extrapolated to long-term mammalian responses.

### 2.2 Neuropsychiatric disorders

Zebrafish display a rich repertoire of evolutionarily conserved, quantifiable behaviors—including analogues of anxiety, depression, social interaction, learning, and locomotion—that serve as functional readouts of CNS activity. Combined with molecular accessibility, this permits simultaneous assessment of signaling pathways and their behavioral consequences, an approach particularly relevant for TCM, which aims to restore integrated physiological and behavioral homeostasis (Table 2).

**TABLE 2:** Zebrafish as an integrative model for TCM in neuropsychiatric research.

Research focus	Representative TCM interventions	Major targets/Pathways	Integrated pharmacological effects	Model limitations
Neurodege-nerative diseases	Tongtian Oral Liquid, Theacrine, Amantadine–Gardenamide A hybrid, Shenghui Decoction, Cistanche tubulosa (via network pharmacology)	Antioxidant enzymes (SOD/GSH-Px/CAT), Sirt3, dopamine-related genes, JNK/p38 MAPK, Aβ/tau pathology, immunomodulatory pathways	Provides integrated neuroprotection through coordinated antioxidant, anti-aggregation, and anti-inflammatory actions, reducing neuronal loss and improving behavioral deficits	1. Differences from humans in brain architecture, drug-metabolizing enzyme systems, and neural substrates of behavior 2. Behavioral phenotypes (e.g., “depression-like”) are not fully homologous to complex human psychiatric conditions. Risk of oversimplification in equating specific behavioral alterations (e.g., reduced locomotion) with specific psychiatric phenotypes. Findings require support from refined behavioral batteries combined with molecular and electrophysiological validation
Epilepsy	Pharbitin (from *Semen Pharbitidis*), coral-derived peptide AdKuz2, *Magnolia officinalis* extracts (magnolol, honokiol), steroidal saponins from *Solanum torvum*, Schaftoside	GABA-glutamate balance, GABAA receptors, multi-mechanistic suppression of apoptosis, inflammation, and oxidative stress	Restores neuronal homeostasis by modulating neurotransmitter balance, receptor activity, and multi-pathway synergy, prolonging seizure latency and suppressing seizures	
Depression and cognitive impairment	Jiawei Xiaoyao Capsule, polyphenols from *Gastrodia elata* (e.g., gastrodin), ethanol extract of *Schisandra chinensis* (schisandrin), extract from *Platycladus orientalis* seeds	Monoamine systems (NE/5-HT/DA), cortisol, tyrosine hydroxylase, RTN4R-mediated neuroinflammation and apoptosis, 5-HT/DA pathways	Exerts antidepressant-like effects and improves cognition via coordinated regulation of monoaminergic transmission, suppression of neuroinflammation/apoptosis, and modulation of arousal states, extending beyond conventional monoamine-centric approaches	
Drug addiction, OCD and stroke	Addiction: Rhynchophylline (Rhy) Stroke/Repair: Guanxinning Tablet (Danshen-Chuanxiong) Toxicity: Aconitine (AC)	Addiction: Dopamine/glutamate systems, miR-181a-5p/GABRA1 axis Stroke: Complement/coagulation cascades, inflammatory networks Toxicity: Serotonin pathways, 5-HT1A receptor	Addiction: Multi-system regulation inhibiting conditioned place preference Stroke Repair: Multi-pathway synergy promoting functional recovery and blood-brain barrier repair Toxicity Assessment: Elucidates neurotoxic mechanisms to inform safe clinical use	

#### 2.2.1 Neurodegenerative diseases

**FIGURE 3:**
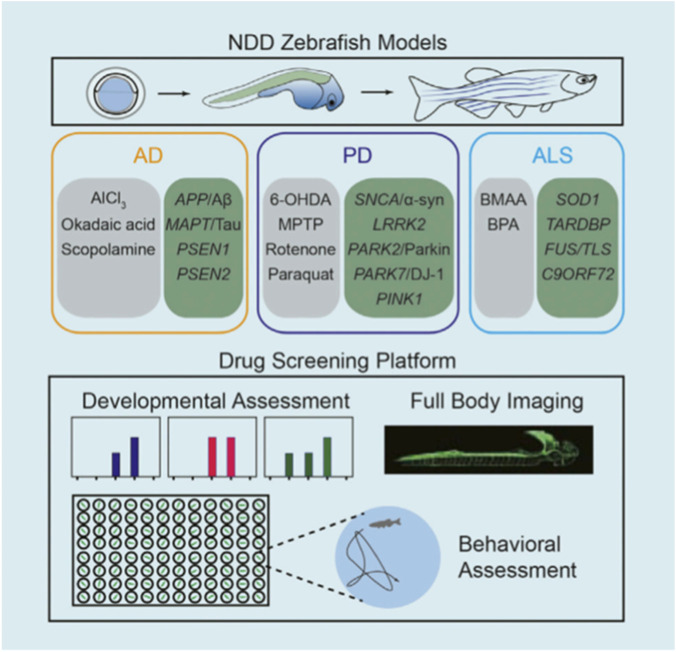
Application of zebrafish for high-throughput drug screening in NDD research (Wang et al., 2021b). This schematic illustrates the application of zebrafish in modeling key neurodegenerative diseases (e.g., AD, PD, ALS), facilitating drug screening, and enabling multi-dimensional phenotyping through developmental assessment, whole-body imaging, and behavioral analysis, thereby bridging molecular mechanisms and organismal-level pathology. Reproduced with permission (Wang et al., 2021b). Copyright © 2021, Frontiers Media S.A.

In models of Parkinson’s disease (PD) and Alzheimer’s disease (AD), TCM interventions have been shown to ameliorate neuronal loss and behavioral deficits through multiple, complementary actions (Figure 3).

In PD models, Tongtian Oral Liquid attenuates MPTP-induced neurodegeneration by scavenging free radicals, elevating antioxidant enzymes (SOD/GSH-Px/CAT), ameliorating mitochondrial dysfunction, and restoring dopamine-related gene expression (Dongjie et al., 2022). Theacrine preserved dopaminergic neurons through Sirt3-mediated reduction of oxidative stress (Duan et al., 2020), and an Amantadine–Gardenamide A hybrid exhibited superior efficacy in reducing dopaminergic neuron loss and α-synuclein aggregation compared to its individual components (Zhu et al., 2023). In addition, Modarresi et al. thoroughly evaluating medicinal plants against PD in zebrafish, highlighting that *Centella asiatica* and Bacopa monnieri demonstrated substantial activity by reducing α-synuclein aggregation and enhancing dopamine synthesis (Chahardehi et al., 2024). While, without side-by-side dose-response and time-course comparisons under the same PD paradigm, it is difficult to judge whether poly-mechanistic interventions genuinely outperform targeted mitochondrial or antioxidant strategies.

In AD models, Shenghui Decoction suppresses JNK/p38 MAPK signaling, resulting in decreased Aβ deposition, tau hyperphosphorylation, neuroinflammation, and synaptic injury (Lu et al., 2024). While a network-pharmacology-guided study identified multiple immunomodulatory compounds in Cistanche tubulosa (Liu et al., 2024a). However, the zebrafish AD models used (typically acute chemical exposure) do not fully recapitulate the chronic, progressive nature of human AD, which limits translational interpretation.

These reports converge on a scenario in which coordinated antioxidant, anti-aggregation, and anti-inflammatory activities contribute to neuroprotection. However, the relative *in vivo* contribution of each mechanism is rarely quantified, and it is often unclear whether the observed behavioral improvements are due to direct neuroprotection or to systemic effects.

#### 2.2.2 Epilepsy

The zebrafish model could effectively links molecular mechanisms (such as neurotransmitter balance and receptor modulation) to whole-organism behavioral seizure phenotypes, which enables the systematic deconvolution of TCM’s multi-target logic in suppressing neuronal hyperexcitability.

Bioassay-guided isolation in zebrafish identified pharbitin from Semen Pharbitidis as a core anti-seizure component in PTZ-induced models (Liu M. et al., 2019). Similarly, the coral-derived Kunitz-type peptide AdKuz2 exhibited potent activity by modulating the GABA-glutamate balance and activating GABAA receptors (Chen H. et al., 2022). Plant extracts, such as magnolol and honokiol from Magnolia officinalis (Li et al., 2020b), steroidal saponins from Solanum torvum (Ren et al., 2024), and Schaftoside (Dang et al., 2021), all prolong seizure latency or reduce seizure severity in PTZ-induced models. The mechanisms invoked span GABA-glutamate balance, apoptosis, inflammation, and oxidative stress. Given that several of these compounds exhibit multi-mechanistic profiles, a key unresolved question is whether the apparent poly-pharmacology genuinely yields additive or synergistic seizure suppression, or whether one primary mechanism (e.g., GABAA receptor potentiation) accounts for most of the efficacy observed.

In summary, the zebrafish model integrates multi-level evidence—from receptor interaction and pathway modulation to behavioral seizure suppression—to dissect how TCM-derived compounds coordinately restore neuronal homeostasis, highlighting its role as a dynamic integrator in anticonvulsant discovery and mechanistic elucidation.

#### 2.2.3 Depression and cognitive impairment

The zebrafish model, with its quantifiable behavioral endpoints and evolutionarily conserved neuroendocrine pathways, exhibit considerable strengths in elucidating the multi-target mechanisms underlying the antidepressant effects of TCM. It effectively bridges molecular interventions—such as neurotransmitter modulation and anti-inflammatory actions—with holistic behavioral and physiological phenotypes, including exploratory activity, stress responsiveness, and sleep-wake regulation.

TCM-derived agents often outperform conventional monoamine-based antidepressants in zebrafish behavioral assays, but this superiority is typically inferred from broader restoration of endocrine rather than from specifically designed comparative experiments. Jiawei Xiaoyao Capsule rescued multiple parameters (exploratory behavior, cortisol, norepinephrine, tyrosine hydroxylase) that sertraline did not (Zhang et al., 2018). Polyphenols from Gastrodia elata, including gastrodin, exerted antidepressant-like effects by inhibiting RTN4R-mediated neuroinflammation and apoptosis (Wang R. et al., 2022). Similarly, ethanol extract of Schisandra chinensis (EESC) and its active component schisandrin induced sedative effects via modulation of 5-HT/DA pathways (Wang et al., 2018). Furthermore, extract from Platycladus orientalis seeds (S4) reduced wakefulness and increased rest duration, effects correlated with modulation of monoaminergic (NE/5-HT/DA) activity (Yan et al., 2022). These findings collectively suggest that TCM interventions engage a wider set of targets, but they also raise the concern that individual behavioral readouts (e.g., reduced locomotion) may be over-interpreted as “antidepressant-like” effects. A shift toward composite behavioral scoring and multi-laboratory replication would increase confidence in these cross-study comparisons.

#### 2.2.4 Drug addiction and obsessive-compulsive disorder (OCD) and stroke

In the addiction field, Rhynchophylline inhibits methamphetamine-induced conditioned place preference via dopamine/glutamate modulation and possibly a miR-181a-5p/GABRA1 axis (Jiang et al., 2016; Zhu et al., 2017; Jiang et al., 2023). While in stroke, Guanxinning Tablet promotes functional recovery through complement/coagulation and inflammatory network regulation (Wang Y. et al., 2024). These mechanistically distinct applications highlight the versatility of zebrafish, but also underscore the difficulty of establishing general principles. Neurotoxic mechanisms, such as aconitine-induced disruption of serotonin pathways via 5-HT1A receptors (Chen et al., 2021) and the biphasic effects of some plant extracts (Savoldi et al., 2017; Braida et al., 2007; de Abreu et al., 2022), further illustrate that dose-response relationships and outcome measures are highly assay-specific.

Owing to its evolutionarily conserved neuroendocrine systems, rich repertoire of quantifiable behavioral paradigms,the zebrafish model successfully bridges a critical gap in research. It provides an empirical research framework for interpreting TCM’s therapeutic philosophy, which emphasizes holistic regulation and the restoration of systemic equilibrium. However, notable differences remain between zebrafish and humans in terms of brain architecture, drug-metabolizing enzyme systems, and the neural substrates underlying certain behaviors. The behavioral phenotypes observed in zebrafish (e.g., “depression-like” or “anxiety-like” states) are not fully homologous to the complex psychiatric conditions in humans. There is a risk of oversimplification in directly equating specific behavioral alterations (such as reduced locomotion) with specific psychiatric phenotypes (such as depression). Therefore, these findings require support from more refined behavioral test batteries combined with molecular and electrophysiological validation.

### 2.3 Metabolic and immune disorders

Zebrafish models offer a transformative window into the integrated pathophysiology of metabolic and immune disorders, a realm where TCM’s holistic principles are prominently applied (Table 3). The model’s paramount advantage lies in its capacity for real-time, *in vivo* visualization of the dynamic crosstalk between metabolic dysfunction and inflammatory processes. This is made possible by optically transparent embryos and a comprehensive toolkit of fluorescent transgenic lines that tag specific cell types—such as hepatocytes, adipocytes, neutrophils, and macrophages. Unlike static endpoint analyses in rodents, zebrafish allow researchers to watch, for instance, how hepatic lipid accumulation dynamically recruits immune cells, or how an herbal intervention like puerarin can simultaneously reduce lipid droplets and modulate macrophage polarization within the same living animal. This direct observation of system-level interactions is pivotal for validating TCM theories like “phlegm-stasis inter-binding”, which posits an inseparable link between metabolic waste and inflammatory stasis.

**TABLE 3:** Zebrafish as an integrative model for TCM in metabolic and immune disorders research.

Research focus	Representative TCM interventions	Major targets/Pathways	Integrated pharmacological effects	Model limitations
Fatty liver diseases (ALD/NAFLD/NASH)	*Pueraria lobata* flavonoids/puerarin, fermented puerarin, Poria Cocos-Pueraria-Hovenia combination, Qigui Jiangzhi Formula, penisterpenoid A, naringin	AMPKα-ACC, alcohol metabolism, autophagy (AMPK-mTOR-ULK1, PINK1/Parkin), macrophage polarization (M2), STAT3/HIF-1α, oxidative stress/apoptosis-related genes (*cyp2y3, fabp10α*)	Ameliorates hepatic steatosis via activating lipid catabolism/autophagy, enhancing antioxidant capacity, modulating gut-liver axis, and promoting anti-inflammatory macrophage polarization, providing multi-faceted protection	Many studies remain at the level of phenotypic correlation and key pathway validation. Future work needs to integrate single-cell omics, spatial metabolomics, and gene-editing to dissect how TCM remodels specific immune cell subsets and modulates inter-organ metabolic-immune crosstalk (e.g., gut-liver-brain axes)
Hyperlipidemia and diabetes	Danggui Shaoyao San, emodin, corn stigma extract, glycitein (from *Lycii Fructus*), ethanol extract of *Morinda officinalis*, salvianolic acid B (Sal B)	PPAR signaling, LDL-C uptake/reverse transport, cholesterol synthesis, sterol biosynthesis pathway, xanthine oxidase (XDH)/urate excretion, MAPK pathway (osteoclast differentiation), ROS scavenging, osteogenic genes	Systemically restores metabolic homeostasis: regulates lipid profiles, controls hyperglycemia, lowers uric acid, and protects against diabetic osteoporosis via anti-resorptive and pro-osteogenic actions	
Inflammatory and immune disorders	Andrographolide (AP-5), gammabufotalin, Betula pendula extract, optimized Liang-Ge-San, Qinggan Yin (QGY), Pueraria polysaccharide (PLP), Gardenia polysaccharide (GJP50–3), Cynanchum paniculatum extract (CP-VE), Artemisia argyi essential oil	MyD88/NF-κB/STAT3, TLR4/MyD88, ROS/COX-2, MAPK pathways, macrophage migration/activity, lymphocyte proliferation, MAPK/NF-κB (gastric repair), MyD88/TRAF6/IL-10 (intestinal repair)	Modulates inflammation/immunity via: 1) precise inhibition of key pro-inflammatory pathways; 2) multi-pathway synergy of compound formulas; 3) immunoenhancement by polysaccharides; 4) repair of mucosal/immune barriers	

#### 2.3.1 Fatty liver diseases (ALD/NAFLD/NASH)

Puerarin exemplifies how a single compound can act on multiple facets of fatty liver disease, activating AMPKα-ACC to reduce steatosis (Liu et al., 2021; Hu et al., 2023). Promoting autophagy via AMPK-mTOR-ULK1, and driving M2 macrophage polarisation (Fang et al., 2024). Fermentation improves puerarin bioavailability and enhances gut barrier function (Liu et al., 2021), raising the question of how much of the hepatic benefit is secondary to intestinal effects. Compared with single-compound interventions, multi-herb combinations such as Poria Cocos–Pueraria–Hovenia (Liu et al., 2021) or Qigui Jiangzhi Formula (Zhang L. et al., 2025) target additional nodes (e.g., neutrophil infiltration, TFEB-mediated autophagy), yet the advantage of these broader interventions has not been rigorously quantified against optimized doses of single agents. Novel compounds such as penisterpenoid A (PINK1/Parkin-mediated mitophagy) (Zhang et al., 2024a) and naringin (cyp2y3, fabp10α downregulation) (Zhou C. et al., 2019) add further mechanistic diversity, but the lack of cross-study standardization of steatosis scoring methods limits direct comparability. Collectively, these studies illustrate how the zebrafish model integrates molecular events—from kinase activation and gene expression to cellular processes like autophagy and polarization—with quantifiable metabolic outcomes.

#### 2.3.2 Hyperlipidemia and diabetes

Metabolic regulation by TCM in zebrafish spans lipid, glucose, and uric acid metabolism, often via overlapping signaling pathways. Danggui Shaoyao San activates PPAR signaling to improve lipid profiles (Wang et al., 2023a), while emodin simultaneously enhances LDL-C clearance and suppresses cholesterol synthesis (He LF. et al., 2022) Corn stigma extract and glycitein demonstrate that distinct chemical scaffolds can modulate sterol and purine metabolism, respectively (Liang H. et al., 2024; Yang S. et al., 2025).

With respect to diabetic complications such as osteoporosis, the ethanol extract of Morinda officinalis counteracted bone loss by suppressing osteoclast differentiation through inhibition of the MAPK pathway (Fu et al., 2020). Additionally, salvianolic acid B rescued dexamethasone-impaired osteogenesis by scavenging ROS (Luo et al., 2016). Although these results suggest that TCM compounds can simultaneously improve metabolic and bone health, the evidence remains largely correlative, and mechanistic studies that directly link target engagement to metabolic endpoints are needed.

#### 2.3.3 Inflammatory and immune disorders

The anti-inflammatory actions of TCM in zebrafish involve both specific pathway inhibition and multicomponent synergy. Andrographolide (AP-5) and gammabufotalin inhibit MyD88-and TLR4-dependent signaling, respectively (Xuemei et al., 2022; Gan et al., 2019; Zheng et al., 2022), whereas compound formulas such as optimized Liang-Ge-San simultaneously suppress MyD88/NF-κB and MAPK pathways (Lu Z. et al., 2021), implying a broader target profile.

Polysaccharides also play key immunomodulatory roles. Polysaccharides from Pueraria and Gardenia enhance macrophage activity and lymphocyte proliferation (Wang H. et al., 2021; Shao et al., 2023) and extracts of Cynanchum paniculatum and Artemisia argyi promote mucosal barrier repair (Wang M. et al., 2025; Meng et al., 2022). Despite this mechanistic plurality, formal comparisons of efficacy and selectivity between individual compounds, defined mixtures, and complex extracts are generally absent, leaving the added value of multicomponent synergy an open question.

**FIGURE 4:**
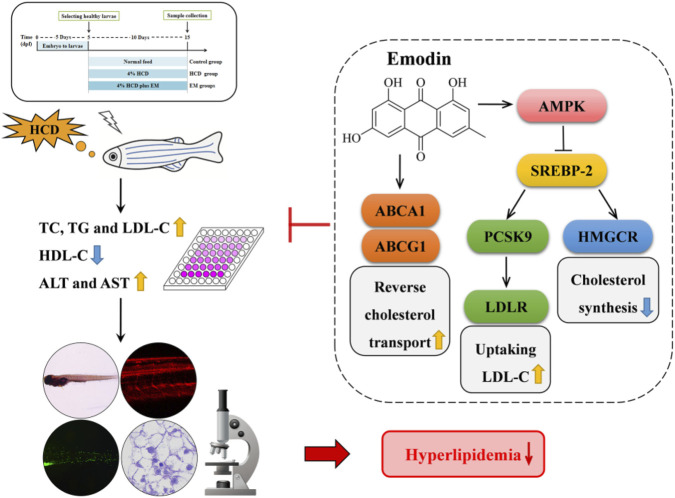
Schematic illustration of the anti-hyperlipidemic mechanism of emodin (EM) in high-cholesterol diet (HCD)-induced hyperlipidemic zebrafish (He LF. et al., 2022). Zebrafish larvae were fed a 4% HCD for 10 days to establish hyperlipidemia (HLP), during which EM was administered at indicated concentrations. This dietary challenge resulted in marked dyslipidemia, as evidenced by elevated total cholesterol (TC), triglycerides (TG), low-density lipoprotein cholesterol (LDL-C), and serum transaminases (ALT/AST), along with decreased high-density lipoprotein cholesterol (HDL-C), excessive lipid deposition in blood vessels and liver, hepatic histological damage, and vascular neutrophil inflammation. Mechanistically, EM activated AMPKα, which subsequently downregulated SREBP-2 to suppress HMGCR-mediated cholesterol synthesis and PCSK9 expression, thereby promoting LDLR-dependent LDL-C uptake; concurrently, EM upregulated ABCA1 and ABCG1 to enhance reverse cholesterol transport. Collectively, these multi-target regulatory effects restored lipid homeostasis and alleviated hyperlipidemia. Reproduced with permission (He LF. et al., 2022). Copyright © 2021, John Wiley and Sons.

The zebrafish model, with its unparalleled capacity for real-time dynamic observation *in vivo*, enables the simultaneous visualization of synchronized processes under TCM intervention, such as hepatic lipid accumulation (Figure 4) coupled with macrophage polarization, hyperglycemia associated with oxidative stress, and the repair of gut microbiota alongside immune barrier restoration. This capability transcends the limitations of static endpoint analyses typical of traditional animal models, providing direct visual evidence for validating TCM’s role in reestablishing metabolic-immune system homeostasis. However, many studies remain confined to phenotypic correlations and validation of key signaling pathways. Future research should integrate single-cell sequencing, spatial metabolomics, and gene-editing technologies to enable a more granular dissection of how TCM remodels the functions of specific immune cell subsets and modulates inter-organ metabolic-immune crosstalk, such as along the gut-liver and gut-brain axes.

### 2.4 Cancer

**FIGURE 5:**
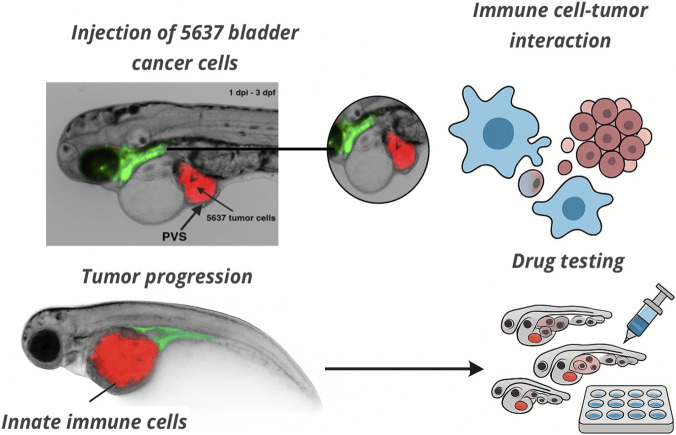
*In vivo* imaging and schematic overview of a zebrafish xenograft model for real-time analysis of tumor–immune interactions and drug screening (Barbosa et al., 2025). Fluorescently labeled 5637 bladder cancer cells (red) were microinjected into the perivitelline space (PVS) of 2 dpf zebrafish larvae, enabling direct visualization of tumor localization and dynamic interplay with host innate immune cells (green). This orthotopic xenograft platform not only permits longitudinal monitoring of tumor progression and immune cell recruitment in living animals, but also supports high-throughput drug screening applications, as functionally illustrated in the schematic workflow. Reproduced with permission (Barbosa et al., 2025). Copyright © 2025, MDPI.

The zebrafish model serves as a critical platform in TCM oncology research, leveraging its unique strengths in high-throughput screening, real-time *in vivo* visualization, and genetic tractability to elucidate anti-tumor mechanisms (Figure 5). To improve clarity and comprehensiveness, this section is organized into two parallel subsections addressing solid tumors and hematological malignancies, respectively (Table 4).

**TABLE 4:** Zebrafish as an integrative model for TCM in oncology research.

Cancer types	Mechanism of action	Key advantages of zebrafish	Major targets/Pathways	Integrated pharmacological effects	Model limitations
Solid tumors	Direct Inhibition of Tumor Progression	High-throughput screening; real-time *in vivo* visualization of tumor cell proliferation, migration, invasion, and metastasis	Induces apoptosis, promotes ferroptosis, arrests cell cycle, reverses epithelial-mesenchymal transition (EMT); core pathways: PI3K/AKT/mTOR, MAPK, Wnt/β-catenin	Multi-target direct suppression of tumor growth and spread, encompassing induction of programmed cell death, proliferation arrest, and metastasis inhibition	1. The adaptive immune system is not fully mature in larvae, limiting evaluation of drugs requiring a complete immune response (e.g., immune checkpoint inhibitors) 2. There is a need to develop transgenic or humanized zebrafish models that more closely resemble the human tumor immune microenvironment
	Remodeling the Tumor Microenvironment (TME)	Visualizes tumor angiogenesis; enables real-time observation of immune cell (e.g., macrophage) dynamics and polarization within tumors	Inhibits VEGF/VEGFR axis (anti-angiogenesis); regulates macrophage polarization (enhances anti-tumor immunity)	Alters the pro-tumorigenic TME through a dual strategy of “starving” the tumor and activating the immune system	
	Improving Chemotherapy Efficacy and Mitigating Side Effects	Facilitates establishment of combination therapy models with chemotherapy; allows assessment of systemic toxicities (e.g., hematopoietic system)	Inhibits autophagy to chemosensitize cancer cells; targets specific signaling molecules; alleviates chemotherapy-induced leukopenia	Serves as chemosensitizers or protective agents, enhancing the efficacy of conventional chemotherapy while reducing its toxic side effects (“synergistic and attenuating”)	
Hematolo-gical malignancies	Transgenic T-ALL (rag2:Myc), AML (AML1-ETO, NUP98-HOXA9), and B-ALL (TEL-AML1, MYC) models; PDX platforms with human AML cells	No immunosuppression needed; Real-time visualization of leukemic dissemination; Low compound volume (ideal for herbal extracts)	PP2A (T-ALL); COX-2/β-catenin, NF-κB (AML) Ubiquitin-proteasome, ferroptosis, glutathione metabolism (RIF); B-lineage vulnerabilities	Restores thymic architecture; induces apoptosis; monitors tumor burden; alleviates hepatotoxicity (RIF); engages non-overlapping anti-leukemic programs vs. ATRA.	Larval adaptive immunity incomplete Short-term observation only (≤7 days) Metabolic differences from mammals

#### 2.4.1 Solid tumors

Solid tumors account for the majority of human cancer morbidity and mortality, and zebrafish models have been extensively leveraged to dissect how TCM interventions impede tumor progression through multi-pronged mechanisms. Current evidence indicates that TCM operates via four interconnected strategic axes in solid tumor contexts.

First, direct cytostatic and cytotoxic effects. TCM agents suppress proliferation, migration, invasion, and metastasis by inducing apoptosis (Deng et al., 2021; Ning et al., 2022; Wang et al., 2020a; Liu et al., 2019b), promoting ferroptosis (Gao et al., 2020; Sun et al., 2023), arresting the cell cycle (LIU et al., 2019b; LI et al., 2016), and reversing epithelial-mesenchymal transition (EMT). These effects are often mediated through modulation of central pathways such as PI3K/AKT/mTOR (Deng et al., 2021; Yang et al., 2025b; Liu et al., 2019c), MAPK (Yang et al., 2025b; Xiao et al., 2022), and Wnt/β-catenin (Huang et al., 2021).

Second, tumor microenvironment (TME) remodeling. TCM remodels the tumor microenvironment through anti-angiogenic effects via inhibition of the VEGF/VEGFR axis (Xu et al., 2024; Liang et al., 2017; Li et al., 2020c; Yang et al., 2023; Zhou et al., 2021; Song et al., 2018; Chen L. et al., 2025; Liu W. et al., 2024; Zou et al., 2020) and by enhancing anti-tumor immunity via regulation of macrophage polarization (Sun et al., 2023; Chen L. et al., 2025; Wang S. et al., 2020).

Third, chemotherapy modulation. TCM agents improve chemotherapy outcomes by sensitizing cancer cells through autophagy inhibition (Wang et al., 2020a; Wang et al., 2019) or by targeting specific signaling molecules (Zhang LW. et al., 2025; Liu Y. et al., 2024), while some formulations mitigate chemotherapy-induced side effects such as leukopenia.

Fourth, delivery optimization. Innovative delivery systems like nanocarriers have been developed to enhance the oral bioavailability and tumor-targeting efficacy of TCM compounds (Zhao et al., 2024; Wang X. et al., 2022; Liu et al., 2025).

Collectively, this evidence validates the efficacy of diverse TCM entities—including compound formulations (e.g., Fangji Huangqi Decoction (Guo et al., 2020), Compound Phyllanthus urinaria L (Huang et al., 2021)), single herb extracts (e.g., Hedyotis diffusa (Ning et al., 2022; Yang et al., 2019), osthole (Chen YQ. et al., 2022), and isolated compounds (e.g., ginsenoside Rf (Chen H. et al., 2025), cryptotanshinone (Fu X. et al., 2021)). Importantly, the zebrafish model elucidates their multi-target mechanisms at the molecular level, providing a robust scientific foundation for the modernization and global integration of TCM.

However, this model still exhibits significant limitations in its application and translation. Firstly, although zebrafish larvae possess a well-developed innate immune system, their adaptive immune system is not fully mature during early developmental stages, which restricts its utility in evaluating drugs that require a complete immune response, such as immune checkpoint inhibitors. Furthermore, zebrafish xenograft models often fail to fully recapitulate the complex tumor microenvironment of human cancers, including intricate stromal components and hypoxic regions. Consequently, there is an urgent need to develop transgenic or humanized zebrafish models that more closely resemble the human tumor immune microenvironment. Overall, the inherent biological differences of zebrafish determine that it serves as an important bridge rather than an endpoint in drug discovery. Its future value depends on our ability to objectively recognize its limitations and effectively integrate it with other models and technologies, thereby jointly advancing the reliable clinical translation of TCM-based anti-tumor research.

#### 2.4.2 Hematological malignancies

In contrast to the spatially confined growth of solid tumors, hematological malignancies disseminate through the circulation–a process that can be directly visualized in the transparent vasculature of zebrafish larvae. Consequently, these malignancies represent a complementary frontier where the zebrafish–TCM interface remains underexplored but technically advantageous. The conserved hematopoietic programs, combined with the feasibility of xenotransplantation without immunosuppression during early larval stages, render this model particularly amenable to high-throughput screening of TCM-derived anti-leukemic agents.

Zebrafish models of T-cell acute lymphoblastic leukemia (T-ALL), acute myeloid leukemia (AML), and B-lineage malignancies provide powerful platforms for studying disease pathogenesis and therapeutic screening. The rag2:Myc transgenic zebrafish develops malignant thymic T-cell proliferation closely resembling human T-ALL and has been used in chemical screens to identify compounds that restore normal thymic architecture and induce apoptosis (Al-Hamaly et al., 2024). For example, perphenazine, which suppresses T-ALL via protein phosphatase 2A (PP2A) activation (Gutierrez et al., 2014), illustrating how phenotypic screening can evaluate TCM-derived alkaloids or related bioactive constituents. For AML, multiple transgenic lines (Harrison et al., 2016) (driven by AML1-ETO, NUP98-HOXA9, MYST3-NCOA2) recapitulate disrupted myelopoiesis and blast accumulation, while patient-derived xenograft (PDX) platforms using human AML cell lines (e.g., MOLM-13) in 48 hpf embryos (Cani et al., 2026) enable real-time monitoring of tumor burden and drug response, particularly suited for TCM formulations acting on COX-2/β-catenin or NF-κB pathways.

A representative TCM application is the Realgar-Indigo naturalis formula (RIF) for acute promyelocytic leukemia (APL). In a zebrafish HL-60 xenograft model, RIF reduced tumor fluorescence and alleviated HL-60-induced hepatotoxicity (fatty vacuolar degeneration). Transcriptomics revealed that RIF modulates the ubiquitin-proteasome system, ferroptosis, and glutathione metabolism–pathways distinct from all-trans retinoic acid (ATRA, which affects FoxO, PI3K-Akt, and apoptosis). Notably, combined ATRA + RIF uniquely engaged autophagosome-lysosome pathways, demonstrating that a single TCM formula can engage non-overlapping anti-leukemic programs compared to standard-of-care ATRA. Additionally, TEL-AML1 (Sabaawy et al., 2006) and MYC-driven B-ALL models (Park et al., 2020) offer systems to study steroid responsiveness and clonal evolution, providing opportunities to test TCM immunomodulatory agents that may synergize with glucocorticoid-based induction therapy or target B-lineage-specific vulnerabilities.

Compared with solid tumor xenografts, leukemia models possess several technical advantages. First, the adaptive immune system is immature during embryonic and early larval stages, which allows robust engraftment of human hematopoietic cells without the need for irradiation or chemical immunosuppression. Second, the transparent circulatory system enables direct, real-time visualization of leukemic dissemination. Third, these models require only small compound volumes, which is compatible with the limited quantities of precious herbal extracts. Future studies should integrate TCM compound libraries with these established leukemia models. Such efforts may accelerate the discovery of novel anti-leukemic agents and deepen our understanding of how multi-component TCM formulae modulate hematopoietic malignancies at the systems level.

### 2.5 Other diseases and pathologies

Beyond the major disease categories, the zebrafish model proves uniquely powerful for investigating TCM bioactivity across a spectrum of other physiological and pathological processes—including tissue regeneration, hepatoprotection, and pigmentation modulation.

In regeneration filed, Rehmanniae Radix Praeparata (Chen F. et al., 2024) and salvianolic acid B (Qin M. et al., 2024) both accelerate fin regrowth, yet the cellular targets (blastema formation, immune cell infiltration, angiogenesis) have been characterized to different depths across studies, making it difficult to compare regenerative potency. In hepatoprotection, corilagin (Wang et al., 2023b) and naringin (Qin et al., 2023) have been shown to modulate macrophage behavior and resolve fibrosis, but the relative importance of direct hepatoprotection and. Immunomodulation is not dissected. Pigmentation studies with germacrone (Li et al., 2024c) and calycosin (Tayier et al., 2021) illustrate how easily scored visual endpoints can screen for pathway modulators, yet the depth of mechanistic follow-up varies substantially among reports. Across these applications, greater standardization of assays and more rigorous quantitative endpoint definitions would significantly strengthen cross-study synthesis and translational relevance.

Furthermore, the model’s adaptability extends to virology and toxicology. It can be used to host pseudoviral infection models for screening antiviral TCM compounds [e.g., against SARS-CoV-2 entry (Lin et al., 2022)],elucidating the cascade of events in toxin-induced injury (e.g., Zearalenone (Luo R. et al., 2025)), and visualizing the interplay between immune dysregulation, oxidative stress, and apoptosis in real time.

In summary, across these diverse applications, the zebrafish model shifts the focus from cataloguing affected molecular targets toward an integrated analysis of restored physiological endpoints. It offers a platform in which the multidimensional and synergistic actions of TCM can be observed and quantified. Whether such actions are aimed at accelerating healing, protecting an organ, or normalizing cellular function, they can be mechanistically interpreted within the context of a living vertebrate. This strategy helps build a rigorous, phenotype-anchored scientific foundation for the therapeutic claims of traditional medicine.

## 3 Safety evaluation and toxicological studies of TCM based-on zebrafish models

**FIGURE 6:**
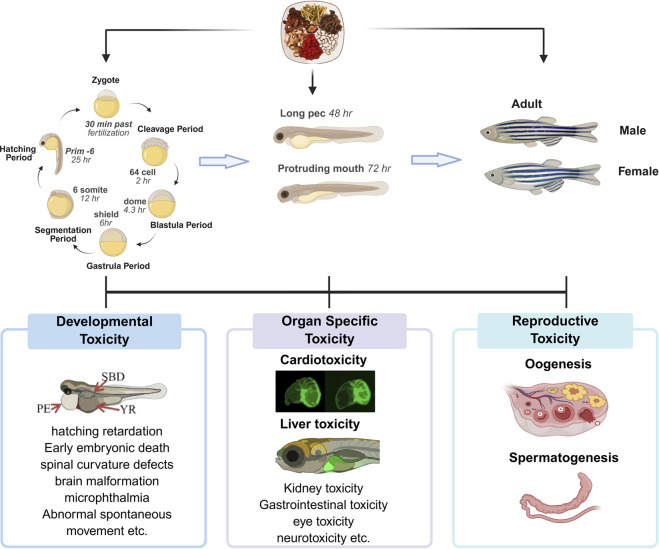
Zebrafish is a powerful model for TCM toxicity evaluation. Schematic illustration of the zebrafish life cycle and its application as an integrated model for multi-dimensional toxicity assessment, including developmental toxicity, cardiotoxicity and reproductive toxicity. This figure was created using BioRender (https://biorender.com) under a valid publication license.

TCM boasts a long history of clinical application, yet research on its safety profiles and toxicity mechanisms remains challenging (Zhang et al., 2022c). Conventional toxicity assessment predominantly rely on mammalian models (e.g., rats and mice), which suffer from limitations such as high costs, prolonged experimental cycles, and ethical controversies. As an emerging model organism, zebrafish offers significant advantages in high-throughput screening and cost-effectiveness (Liu et al., 2020). A single experiment can evaluate hundreds of compounds at a cost approximately 1/1000 of mammalian models (Liu et al., 2020). Its transparent embryonic development enables real-time observation of organogenesis and toxicological phenotypes (Liu et al., 2017; Liu et al., 2024d; Lu et al., 2020) (e.g., pericardial edema, spinal curvature defects). Furthermore, the zebrafish possesses metabolic systems highly conserved in mammals, effectively simulating the metabolic processes of herbal compounds *in vivo* (He et al., 2012; Quan et al., 2019; Giselbrecht et al., 2022). They also align with the 3R principles (Replacement, Reduction, Refinement), making them suitable for early-stage toxicity screening (Hughes and Hessel, 2024; Hillman et al., 2024). In recent years, zebrafish models have been extensively utilized in toxicity screening and mechanistic studies of TCM (Figure 6). Current toxicological investigations using zebrafish primarily focus on developmental, organ-specific, and reproductive toxicity.

### 3.1 Developmental toxicity

Developmental toxicity assessment systems primarily encompass two critical stages: the embryonic and larval periods. Embryonic evaluation focuses on morphological indicators, including hatching rate, somite formation, heart rate, and teratogenicity rates (e.g., pericardial edema, yolk sac cysts), combined with median lethal concentration (LC50) and sub-lethal concentrations (LC10/LC25) for toxicity classification (Wang et al., 2020c). Notably, the chorionic membrane may impede drug permeation, necessitating manual removal or optimized exposure time windows to enhance detection accuracy (Macrae and Peterson, 2015; Wang et al., 2017). Larval-stage assessments emphasize organ-specific toxicity and behavioral analyses: hepatotoxicity is quantified via hepatic grayscale values, area changes, and fluorescence intensity in transgenic lines (Qin M. et al., 2024), while neurotoxicity is evaluated through locomotor activity, circadian rhythms, and stress responses (Gu et al., 2023; Kalueff et al., 2013). Tripterygium wilfordii and its monomers (triptolide and celastrol) significantly inhibit embryonic swimming distance at exceptionally low concentrations (0.001 μg/mL), indicating developmental neurotoxicity (Romero et al., 2023). 5-Hydroxymethyl-2-furfural (5-HMF) induces skeletal maldevelopment, reduced bone mineralization, and behavioral deficits via ROS generation, with toxicity partially reversible by the antioxidant NAC (Jiang et al., 2022). Green tea extracts and the active constituent EGCG (0.01–0.02 μg/mL) trigger sustained hyperactivity, providing the first evidence of their developmental neurotoxicity (Romero et al., 2023). Cigarette smoke extract (CSE) compromises DNA repair, induces aberrant apoptosis, and disrupts lipid metabolism, leading to reduced hatching rates and increased head/heart malformations (Chen J. et al., 2024). Croton tiglium seed aqueous extract (AECT) promotes oxidative stress and apoptosis, causing significant developmental and genotoxic effects (Yumnamcha et al., 2022). Geniposide elevates ROS levels and activates the mitochondrial apoptotic pathway (Bax/Bcl-2 imbalance, Caspase-3 activation) to exert toxicity (Xia et al., 2021). Collectively, these studies establish ROS-mediated oxidative stress, DNA damage, and dysregulated apoptosis as unifying mechanistic pathways underlying developmental toxicity.

### 3.2 Organ toxicity

The zebrafish model plays a pivotal role in mechanistic studies of organ toxicity, particularly for hepatotoxicity evaluation. Seven components from Tripterygium wilfordii (e.g., triptotriterpenic acid A for direct hepatotoxicity and triptobenzene H for macrophage-mediated indirect hepatotoxicity) induce liver injury via distinct pathways (Li M. et al., 2023). Isoliquiritigenin activates the ER stress-UPR pathway (Hu et al., 2024), leading to protein misfolding and apoptosis. Phytolaccagenin triggers hepatic injury through ferroptosis, lipid metabolism dysregulation, and inflammation (Cui et al., 2025). Zuotai induces hepatocyte apoptosis, with toxicity not attributed to its primary component HgS (Zhou LL. et al., 2019). Toosendanin causes liver damage via the IL-1β/MyD88/p65 inflammatory axis and autophagy-apoptosis crosstalk (Sun et al., 2021). Euphorbia kansui disrupts amino acid/energy metabolism while activating oxidative stress and apoptotic pathways (Zhao et al., 2019). Technologically, transgenic lines (such as LFABP:EGFP) and integrated multi-omics (transcriptomics/metabolomics) have significantly advanced mechanistic insights (Li M. et al., 2023; Jia et al., 2020; Han et al., 2024). For cardiotoxicity, diterpenoid alkaloids with C-8 β-acetylated diester structures (e.g., AC, MAC) exhibit potent toxicity, whereas monoester types (e.g., BAC) show weaker effects (YE et al., 2021). Aconitine induces pericardial edema via calcium signaling dysregulation (L-type calcium channel/RyR2 disturbance) and the p38MAPK apoptotic pathway (Li M. et al., 2020). High concentrations of Tanshinone IIA suppress cardiac development, with dechorionated embryos displaying enhanced sensitivity (Wang et al., 2017). In neuro/renal toxicity studies, cinnamaldehyde causes brain structural damage through oxidative stress (reversible by astaxanthin) (Chang et al., 2022), while aristolochic acid (AA) induces renal cystic dilation via disrupted oxidative phosphorylation and ER stress pathways (Chen M. et al., 2023). Modarresi Chahardehi et al. comprehensively reviewed how zebrafish embryotoxicity tests enable quantitative assessment of organ-specific toxicity using standardized scoring systems, linking morphological defects such as pericardial and yolk sac edema to underlying mechanisms (Chahardehi et al., 2020).

### 3.3 Reproductive toxicity

Reproductive and sex-specific toxicity represents an emerging research focus, with zebrafish models elucidating critical mechanisms: Psoralen (BV) binds to the endoplasmic reticulum chaperone BiP and estrogen receptor ESR1, activating the ER stress-UPR pathway to specifically induce follicular atresia and oocyte apoptosis in female zebrafish (Huang et al., 2023). Houttuynia cordata inhibits the PI3K-Akt signaling pathway, resulting in embryonic apoptosis and developmental arrest (Liu Y. et al., 2023). Emodin-8-O-β-D-glucoside (Em8G) exhibits sexually dimorphic toxicity—disrupting the tricarboxylic acid (TCA) cycle in males while predominantly impairing amino acid metabolism in females (Han et al., 2024). These integrated approaches highlight critical mechanisms and risk-benefit trade-offs in TCM reproductive safety assessment.

While zebrafish models offer significant advantages in toxicity studies of TCM, several challenges remain unresolved. First, model validation and standardization require urgent improvement due to the complexity of TCM components and the lack of unified dose-conversion criteria (Liu et al., 2017), particularly in harmonizing evaluation metrics across studies. Second, inconsistent stability and reproducibility (Liu et al., 2017) in certain experimental setups compromise the reliability and cross-study comparability of results. Third, deeper integration of multi-omics approaches—including metabolomics, transcriptomics, and advanced imaging technologies, which is essential to comprehensively dissect toxicity mechanisms, bridging current gaps in mechanistic elucidation. Addressing these challenges will enhance the translational value of zebrafish models in TCM safety assessment.

Zebrafish toxicity studies are most powerful when they shift from cataloging LC50 values to constructing mechanism-toxicity relationships. For example, identifying that hepatotoxicity of Herb X is consistently linked to CYP450 inhibition across studies provides a predictive framework for assessing related compounds. This transforms zebrafish from a simple toxicity filter into a platform for building read-across models and understanding the fundamental biochemical vulnerabilities that underlie TCM’s narrow therapeutic windows, directly informing safer clinical use and formula design.

## 4 Strategies to establish zebrafish models for TCM study

### 4.1 Disease categories

#### 4.1.1 Models in modern medical diseases

Disease models in modern medical research correspond directly to specific pathological states and serve as a fundamental basis for screening drug efficacy and assessing toxicity. In cardiovascular disease models, gene editing (e.g., Cas9/sgRNA) or high-cholesterol diets (Lin et al., 2024; Deng et al., 2024; Vasyutina et al., 2022) are often used to establish atherosclerosis models for evaluating traditional Chinese medicines that promote blood circulation and remove stasis, with key measurements including vascular plaques, blood lipid levels, and relevant gene expression. Liver disease models (Wakai et al., 2024; Zhang et al., 2019) frequently employ chemical agents such as isoniazid to induce hepatic injury for assessing hepatoprotective drugs or hepatotoxicity, analyzed through liver morphology, apoptosis, and enzymatic indicators. Neurological models (Hong et al., 2024; Maciag et al., 2020; Li X-H. et al., 2025) can be constructed via chemical induction or specific genetically modified strains to study anxiolytic effects, memory enhancement, or neurotoxicity. Inflammation models commonly use copper sulfate (CuSO_4_) (Singh et al., 2022; Szumlak et al., 2024) or tail sectioning (Leiba et al., 2023; Arroyo et al., 2024; Denans et al., 2022) to induce acute inflammation for anti-inflammatory drug screening, evaluated by inflammatory cell migration and related cytokine gene expression. Furthermore, leveraging the strong regenerative capacity of zebrafish, tissue regeneration models based on tail fin amputation (Chen F. et al., 2024) are utilized to screen drugs that promote regeneration, primarily monitoring regeneration length, speed, and the dynamics of cell proliferation and apoptosis.

#### 4.1.2 Models in TCM syndrome

TCM syndrome models aim to simulate the holistic characteristics of TCM syndromes and represent a distinctive tool in TCM research. For instance, a “qi deficiency” and blood stasis syndrome model can be established in zebrafish, manifesting as reduced locomotor capacity and declined cardiac function, which can be ameliorated by drugs such as Naoxintong Capsule (Hu et al., 2021). The blood stasis syndrome model is often induced by cold or ice-water stimulation combined with adrenergic agents (Ling and Xu, 2013), evaluated through indicators such as blood circulation velocity, thrombus formation, and hemorheological parameters. Furthermore, other common syndrome models include glucocorticoid-induced kidney “yang deficiency” (Ling and Xu, 2013) assessed by reproductive function, energy metabolism, and relevant hormone levels, as well as models simulating “yin deficiency” or “spleen deficiency” through pharmacological or environmental stress approaches (Xu et al., 2022), thereby enabling holistic evaluation of the regulatory effects of Chinese medicinal interventions.

### 4.2 Gene editing models

Compared to traditional zebrafish models that primarily rely on chemical induction or physical injury to simulate disease phenotypes, focusing on phenotypic observation and preliminary efficacy screening, gene-edited zebrafish models utilize technologies such as Morpholino, CRISPR/Cas9, base editors, and prime editors to achieve targeted gene knock-down/out, knock-in, and even point mutations (Table 5) This enables the high-fidelity modeling of human diseases at the genetic level. In the modernization of TCM research, this model provides a powerful *in vivo* validation platform. Its core value lies in directly linking pharmacological effects with gene functions, primarily through the following four logically progressive approaches:

**TABLE 5:** Comparison of gene editing technologies for TCM research.

Function category	Representative technology	Editing efficiency and Off-target risk	Advantages for TCM research	Challenges for TCM research
Knockout (KO)	CRISPR/Cas9, TALENs	High efficiency, Moderate off-target risk	Rapid, cost-effective models for high-throughput compound screening (Uribe-Salazar et al., 2022)	Uncontrolled mutations; unsuitable for precise disease modeling
Base Editing (BE)	Adenine Base Editor (ABE), Cytosine Base Editor (CBE)	Very high on-target efficiency, Low off-target risk	Ideal for creating precise SNP models to study therapeutic correction (Qin et al., 2024b)	Limited to base transitions; potential for bystander edits
Precise Knock-in/Replacement	Homology-Directed Repair (HDR), Prime Editing (PE)	Moderate-High efficiency, Very low off-target risk	Enables versatile, precise edits for sophisticated mechanistic studies (Vanhooydonck et al., 2025)	Complex design; lower efficiency for large insertions

Firstly, it validates targets predicted by network pharmacology. By knocking out genes identified as potential TCM targets through bioinformatics prediction, the necessity of these targets can be reversely verified *in vivo* (He S. et al., 2022). For instance, if the blood-activating and stasis-resolving effect of Salvia miltiorrhiza is predicted to be associated with genes like VEGF, administering its extract to zebrafish with the corresponding gene knockout would indicate the target’s essential role if the pro-angiogenic effect is abolished.

Secondly, it elucidates the synergistic mechanisms of TCM compound formulations. For complex formulas, knocking out core pathway genes associated with individual component herbs allows researchers to observe changes in the overall formula’s efficacy (Lu Z. et al., 2021; Rao et al., 2024). This helps determine whether the herbs act through identical, parallel, or complementary signaling pathways, thereby clarifying the scientific rationale behind formula compatibility at the molecular level.

Thirdly, it explores the modern biological basis of “treatment based on syndrome differentiation”. By editing genes potentially related to specific TCM syndromes, models with different intrinsic pathological states can be constructed. This enables the scientific study of “treating the same disease with different methods” or “treating different diseases with the same method”. For example, observing the differential efficacy of the same formula on models simulating “yang deficiency” versus “yin deficiency” can provide a molecular explanation for this core TCM principle.

Finally, it deepens the safety evaluation of TCM. By knocking out key genes involved in drug-metabolizing enzymes (e.g., CYP450 family) or toxicity-sensing pathways (Zhang et al., 2022c; Li et al., 2026), genetically susceptible individuals can be modeled. This facilitates in-depth study of the mechanisms behind TCM component toxicity and individual variation, providing precise early warnings for clinical safety.

In summary, the gene-edited zebrafish model, through the research paradigm of “constructing precise models—administering drug interventions—validating gene functions”, successfully translates TCM-related questions—such as target prediction, formula logic, the concept of syndrome differentiation, and safety concerns—into scientific inquiries that can be precisely manipulated and observed within a living organism. This represents a profound shift from correlation to causality and from “phenotypic screening” to “mechanistic elucidation”, establishing the model as a critical bridge connecting traditional TCM theory with modern life sciences. However, the application of gene-edited zebrafish models still faces significant challenges spanning both fundamental technical limitations and the complexities of aligning these tools with the theoretical framework of TCM research.

At the technical level, multiple bottlenecks exist in model construction and characterization. Firstly, the precision and efficiency of the gene-editing process itself are primary constraints. Precise genetic manipulations, such as knock-in to model human single nucleotide polymorphisms (SNPs), are considerably less efficient than gene knockout and carry an inherent, difficult-to-eliminate risk of off-target effects (Prykhozhij and Berman, 2024; Zheng et al., 2025). Secondly, the technology lacks refined spatiotemporal control (Hu et al., 2025; Sui et al., 2025; Zhuo et al., 2021). The current absence of mature and efficient inducible or tissue-specific editing systems makes it challenging to simulate the dynamic processes by which TCM interventions might act on specific organs or at particular stages of a disease. Furthermore, even after successfully generating a genetic model, a significant bottleneck remains in conducting high-throughput, standardized deep phenotyping (Whyte-Fagundes et al., 2025; Sarapultsev et al., 2025a) — assessing complex behaviors, systemic metabolism, and other integrated parameters. This limitation hinders the comprehensive, multi-dimensional evaluation of the holistic efficacy of TCM interventions.

Applying these technological models to TCM research presents deeper challenges related to the integration of distinct scientific and philosophical logics. The most prominent contradiction lies in the fact that gene editing excels at creating “disease models” based on alterations to one or a few genes, whereas a TCM syndrome is fundamentally a complex, interactive network of functional states involving multiple genes and physiological systems (Zhang et al., 2013; Xu et al., 2025). This creates a gap in their foundational philosophies and representational dimensions. This gap directly leads to a second major challenge, which is that TCM formulas typically exert their effects through a synergistic “multi-component, multi-target” network. In a single-gene knockout model, the therapeutic effect of a TCM formula might be preserved through alternative or compensatory pathways, thereby rendering the traditional causal inference logic based on verifying “necessary targets” inadequate for fully explaining the holistic mechanisms of TCM (Yang et al., 2025c; Peng et al., 2025). Finally, standardization and reproducibility of experiments pose a serious practical challenge. Variability in gene-editing efficiency (Siddiqui et al., 2025), the genetic background of zebrafish lines (Martinez-Bautista et al., 2024), and differences between batches of TCM extracts (Yang L. et al., 2025; LI X. et al., 2023) can all compromise the consistency and comparability of findings across different studies.

### 4.3 Drug delivery methods

In zebrafish model-based TCM research, various drug administration methods are employed in a complementary manner, tailored to specific research objectives and developmental stages (Table 6). Embryonic immersion serves as the foundational technique for high-throughput screening (White et al., 2016). This method is primarily applicable to embryonic and larval stages (0–7 days post-fertilization, dpf), where compounds dissolved in the rearing water are absorbed through the skin and gills. Its operational simplicity and high-throughput capacity make it widely used for the preliminary screening of active ingredients, efficacy evaluation (e.g., observing the effects of Salvia miltiorrhiza extract on angiogenesis (Chen J. et al., 2022; Liang Q. et al., 2024)), and multi-organ toxicity safety assessments.

**TABLE 6:** Comparison of drug delivery methods in zebrafish for TCM Research.

Methods	Applicable stage	Key advantages	Major limitations
Embryonic immersion	Embryo/Larva (0–7 dpf)	Simple, high-throughput; ideal for initial bioactivity screening and toxicity assessment	Passive diffusion; limited by compound solubility, permeability, and stability; no spatiotemporal control
Microinjection	Early Embryo (e.g., 1-4 cell)	Precise spatiotemporal control; allows targeted delivery and PK/PD tracing; bypasses absorption barriers	Technically demanding, low-throughput; risk of physical damage; not suitable for chronic administration
Oral gavage	Larva/Adult	Mimics clinical oral route; suitable for studying GI absorption, metabolism, and oral bioavailability	Technically challenging and time-consuming; can cause stress/injury; limited by fish size
Topical administration	Larva/Adult	Simple; useful for studying dermal permeability and evaluating local pharmacological effects (e.g., wound healing)	Limited application scope; difficult to quantify systemic absorption
Nano-Carrier delivery (e.g., polymeric NPs, liposomes, SNEDDS)	All stages (via immersion, injection, or gavage)	Enhances solubility, stability, bioavailability, and targeting of TCM compounds; enables co-delivery and visual tracking (e.g., fluorescent); demonstrates synergistic effects and reduced toxicity	Complex carrier design; challenges in multi-component loading and controlled release; requires further translational validation from zebrafish to mammals

For studies requiring precise spatiotemporal control, such as targeted delivery or pharmacokinetic tracing, microinjection is utilized. This technique involves direct delivery of agents into specific sites during early embryonic stages (Weissenboeck et al., 2025; Guo et al., 2025). To better mimic clinical administration routes and study gastrointestinal absorption and metabolism, oral gavage is applied (Su et al., 2025). Furthermore, topical administration is employed for investigating dermal permeability (Morikane et al., 2020) or local pharmacological effects, such as wound healing (Naomi et al., 2021; Vaidyanathan and Lokeswari, 2024). These methodologies are mutually reinforcing, collectively supporting in-depth TCM research across multiple dimensions, including bioactive discovery, safety evaluation, and mechanistic elucidation.

However, natural active compounds derived from TCM often suffer from poor water solubility, low bioavailability, inadequate stability, and weak targeting capability, which significantly restrict their clinical application (Fu et al., 2024; Yuan et al., 2024). Nanonization techniques and nano-delivery systems can markedly enhance the solubility, stability, bioavailability, and targeting efficacy of these bioactive components. For instance, polymer nanoparticles (e.g., PLGA) loaded with curcumin (Singha et al., 2024) exhibit superior pro-angiogenic activity and reduced developmental toxicity in zebrafish models compared to the free drug. Liposomes, through surface modification for active targeting, have been visualized via fluorescent tracing (Liu YS. et al., 2023) to accumulate specifically in tissues like the intestine, providing direct evidence for elucidating TCM action sites. Self-nanoemulsifying drug delivery systems (SNEDDS) composed of natural lipids can efficiently co-deliver curcumin and piperine (Kazi et al., 2023), and their synergistic effects and favorable biosafety have been validated in zebrafish embryos. These studies collectively demonstrate that nanotechnology can significantly improve the therapeutic index of TCM constituents.

Despite the promising prospects, this field faces several challenges. Technically, the design of nanocarriers requires a delicate balance between targeting specificity, prolonged circulation, and controlled release, while also addressing the complexity of co-delivering multiple TCM components (Zhang YB. et al., 2024). In terms of model translation, further investigation is needed to bridge the gap between findings in zebrafish and their relevance to mammalian systems and clinical outcomes (Patton et al., 2021). Future advancements will depend on the deep integration of materials science, pharmaceutics, and biological methodologies. The development of more intelligent nano-delivery systems, combined with gene-editing technologies in zebrafish, holds the potential to systematically elucidate and optimize precision therapeutic strategies for TCM at both molecular and organismal levels.

## 5 Remaining challenges

Despite notable progress in pharmacological evaluation, bioactive screening, and toxicological assessment using zebrafish, the translational path of TCM faces persistent challenges. Some of these challenges stem from the nature of TCM itself, while others arise from inherent limitations of the zebrafish platform. Critically, the two sets of limitations often intersect: the model’s biological constraints can amplify existing knowledge gaps in TCM research, and the lack of quantitative, cross‐species frameworks makes extrapolation to human conditions uncertain. The following sections address these intertwined challenges, focusing on areas where the zebrafish model falls short of fully bridging TCM theory and clinical reality.

### 5.1 Unclear mechanisms of multi-component synergy

TCM formulae are designed to act throughmulti-component, multi-target coordination, yet the individual contributions and interactions of constituents remain poorly defined. For instance, Gansui Banxia Decoction modulates JAK-STAT and other pathways to alleviate hepatocellular carcinoma ascites, but the specific role of each ingredient is not quantifiable (Feng et al., 2021). Similarly, although Lingjiao Gouteng Decoction demonstrates efficacy in Parkinson’s disease models, it is unclear which components drive the reported anti-inflammatory, antioxidant, or mitochondrial protective effects, or whether they act synergistically (Ni et al., 2023).

From a zebrafish perspective, the common practice of immersion exposure in embryos or larvae adds further complexity. Drug uptake, tissue distribution, and metabolism are difficult to control and measure in these small organisms, making it challenging to establish quantitative exposure–response relationships for individual compounds and their combinations (Guarin et al., 2021a; Grasse et al., 2024; Nawaji et al., 2024). Moreover, zebrafish drug-metabolizing enzymes differ from their mammalian orthologs in substrate specificity and ontogeny (Verbueken et al., 2018), so metabolic activation or detoxification observed in zebrafish may not directly translate to humans (Saad et al., 2017). Consequently, mechanistic insights gained from zebrafish models risk overestimating or underestimating the degree of synergistic interaction (Van et al., 2020; Parthasarathy et al., 2025), unless they are critically calibrated against mammalian pharmacokinetic data.

### 5.2 Low delivery efficiency of active TCM components

Many active TCM components (e.g., flavonoids) suffer from poor water solubility, low oral bioavailability (often below 10%), and short *in vivo* half-lives (Wei et al., 2022; Qiu et al., 2023). Biological barriers such as the blood-brain barrier further restrict delivery, with brain concentrations of gastrodin and paeoniflorin reported at only 1/20 to 1/30 of plasma levels (Wei et al., 2022; Yang et al., 2024). These delivery shortcomings not only necessitate higher doses but also increase the risk of off-target systemic effects.

Zebrafish models introduce an additional layer of uncertainty in pharmacokinetic extrapolation (Lu et al., 2025). Immersion administration bypasses first-pass metabolism and permits direct absorption through the skin and gills, which differs substantially from oral dosing in mammals (Morikane et al., 2020; Guarin et al., 2021b). Even when oral gavage is applied in adult zebrafish, gastrointestinal transit, enteric metabolism, and transporter-mediated absorption can diverge significantly from mammalian physiology (Acuff and Guillemin, 2024; Erradhouani et al., 2024). As a result, tissue exposure profiles measured in zebrafish often cannot be directly scaled to predict human pharmacokinetics. This disconnect weakens the model’s ability to guide formulation optimization or dose selection in early-stage development (Tan et al., 2026).

### 5.3 Lack of objective and quantifiable diagnostic criteria

TCM diagnosis relies heavily on pattern differentiation (“Bian Zheng”),which involves subjective judgment (e.g., tongue and pulse diagnosis) and shows inter-practitioner consistency often below 60% (Duan et al., 2021). Modern omics technologies have not yet been systematically integrated to establish reproducible correlations between TCM patterns and molecular biomarkers (Wen et al., 2025), which hampers the alignment of efficacy evaluation with evidence-based medicine.

Here, the zebrafish model faces a parallel translational gap. Behavioral and physiological phenotypes observed in zebrafish are not exact homologues of human clinical symptoms (Racca et al., 2026; Costa et al., 2023). For example, a reduction in locomotion in zebrafish is frequently interpreted as “depression-like” or “anxiety-like” behavior, yet such single-parameter readouts cannot capture the complexity of human psychiatric conditions (Yang B. et al., 2025). While the fish may display measurable responses to TCM intervention, equating these responses with relief of a specific human syndrome risks oversimplification (Costa et al., 2023). The absence of validated, cross-species phenotypic equivalences limits the utility of zebrafish data in constructing objective diagnostic or therapeutic response criteria for TCM (Racca et al., 2026).

### 5.4 Deficiencies in clinical trial design and evaluation systems

TCM clinical research has been criticized for small sample sizes, lack of randomization and blinding, and insufficient multicenter collaboration (Yang et al., 2024; Wen et al., 2025). Evaluation standards also diverge. Western medicine prioritizes tumor shrinkage or biomarker change, whereas TCM emphasizes restoration of “healthy qi” and quality of life - a discrepancy that complicates international acceptance (Fu R. et al., 2021).

Zebrafish experiments themselves often represent a form of acute screening rather than longitudinal disease modeling. Most pharmacological studies in zebrafish span hours to a few days, using embryonic or larval stages. This short time-frame cannot replicate the chronic, progressive nature of many human diseases for which TCM is indicated (Racca et al., 2026). Moreover, the adaptive immune system of zebrafish larvae is not fully mature, which precludes evaluation of treatments that depend on coordinated innate and adaptive immunity, including many anti-cancer strategies such as immune checkpoint inhibition (Barbosa et al., 2025). Without a mature immune system and extended observation periods, the model cannot fully assess long-term efficacy or safety outcomes that are central to clinical trial design (Sarapultsev et al., 2025b; Bangeppagari et al., 2025).

### 5.5 Lack of unified standards for herb quality and processing

The quality of Chinese medicinal herbs is highly variable greatly due to geographic origin, processing methods, and adulteration. For example, improper steaming can reduce gastrodin content by 40% (Zhu et al., 2022). Clinical substitutions further undermine therapeutic consistency (Yang et al., 2024).

This variability directly impacts zebrafish research. Many studies use herbal extracts without detailed chemical characterization, making it difficult to compare results across laboratories (Gence et al., 2025). Differences in zebrafish genetic background, rearing conditions, and administration protocols further compromise reproducibility. Without standardized extract chemistry and validated experimental frameworks, the zebrafish model cannot reliably discriminate between genuine biological signals and batch-specific artifacts (Ngu et al., 2025). This reproducibility bottleneck undermines the model’s credibility as a translational platform.

## 6 Future perspectives

Facing core challenges in the modernization of TCM—such as the difficulty in holistically elucidating the “component–mechanism–efficacy” relationship, the lack of a standardized biological basis for clinical experience, and the absence of evaluation models for complex systems—future development will closely rely on dynamic *in vivo* integrated systems like the zebrafish model. This approach aims to establish an innovative research paradigm capable of bridging molecular mechanisms with holistic effects. Leveraging its high-throughput capacity, visualizability, compatibility with systems biology, and high evolutionary conservation with humans, the zebrafish model is poised to become a key engine for deciphering the scientific connotation of TCM’s holistic perspective.

### 6.1 AI-driven multi-omics to decipher formulae synergy networks

The current challenge lies in the absence of an effective bridge for living integration and functional validation between massive multi-omics data and the complex chemical system of TCM. The integration of AI-based modeling with zebrafish phenomics offers a practical route to begin addressing this gap. Recent work has already demonstrated the feasibility of this approach. For example, an AlphaFold-based AI docking study combined with a zebrafish model of metabolic-associated fatty liver disease identified the AMPK/SIRT1-TFEB pathway as a target of a multi-herb formula and confirmed its ability to reduce hepatic lipid accumulation (Zhang L. et al., 2025). This study illustrates how computational prediction and *in vivo* phenotypic screening can be linked in an iterative cycle - the AI model proposes a component-target-pathway network, and zebrafish assays provide the data to refine these predictions. Zebrafish embryos and larvae are particularly suitable for generating the coherent, high-dimensional datasets required to train such models. Systematic perturbation of individual formula components, combined with CRISPR-based gene editing and real-time imaging of pathway activities (such as PINK1/Parkin-mediated mitophagy (Moskal et al., 2023)), which can produce data layers that relate chemical input to tissue-specific gene expression and phenotypic outcome. Li et al. showed that BSTSF alleviated AD pathology by differentially regulating cysteine/methionine metabolism in the cortex and glutamine/glutamate metabolism in the hippocampus (Li et al., 2022), exemplifying the brain-region-specific mechanistic data essential for training future AI models. AI frameworks trained on these multi-parametric *in vivo* profiles may then start to resolve the functional contributions of each ingredient, ultimately translating the abstract “sovereign-minister-assistant-courier” principle into a set of experimentally testable and quantitatively defined interaction maps.

Nonetheless, such studies are at a preliminary stage and require further development. Most AI models to date have been trained on mammalian or cell-line datasets and require careful cross-species calibration before they can be confidently applied to zebrafish data. Furthermore, the chemical space of TCM is far larger than the number of compounds that have been systematically profiled in zebrafish. Closing the loop from *in silico* prediction to *in vivo* confirmation therefore depends on sustained expansion of annotated zebrafish phenomic datasets and on rigorous benchmarking of model performance against known pharmacological standards. In this context, zebrafish is not yet a mature engine for AI-driven TCM discovery, but it is a uniquely positioned testbed for developing and validating the cross-species, multi-scale models that the field urgently needs.

### 6.2 Developing humanized models for systemic functional evaluation

Traditional efficacy evaluation models often fail to replicate the complex interactions within the human body, such as the gut–liver axis and immune–microbiome crosstalk, which are inadequate for assessing the holistic regulatory effects of TCM on multi-organ systems (Hua et al., 2025; Wu et al., 2025; Luo W. et al., 2025). The development of humanized chimeric models in zebrafish, combined with systemic functional indices, represents a promising breakthrough. By transplanting patient-derived organoids into zebrafish, it is possible to study human hepatic metabolism of TCM components (Shimizu et al., 2023), their modulation of engineered intestinal barriers and microbiota (Rawling et al., 2023), as well as personalized anti-tumor effects (Fazio et al., 2020) — all within a complete living circulatory and immune microenvironment.

A further significant advance lies in leveraging the zebrafish’s transparent body and fully developed multi-organ systems to establish a multi-parameter (Vieira et al., 2025) “Systemic Functional Recovery Index”. This index integrates cardiac function, vascular perfusion, renal filtration, neural behavior, energy metabolism, and other key physiological parameters. It enables dynamic and quantitative assessment of the recovery of whole-body homeostasis following TCM intervention (Chen S. et al., 2022), thereby offering an unprecedented holistic and real-time quantitative tool for evaluating TCM concepts such as “harmonizing yin and yang” and “reinforcing vital qi while eliminating pathogenic factors”.

### 6.3 Innovating nano-delivery systems to enhance targeting and efficacy

To address the delivery bottlenecks of TCM components, intelligent nano-delivery systems based on TCM polysaccharides (which serve dual roles as carriers and therapeutics), liposomes (Alizadeh et al., 2025; NI et al., 2025), polymeric nanoparticles (Zhai et al., 2024), mesoporous silica (Lin et al., 2019), and other materials should be vigorously developed (Wei et al., 2022; Chai et al., 2025; Liu Z. et al., 2023; Zheng et al., 2019; Meena et al., 2020) These systems can significantly improve the solubility and stability of poorly soluble components, achieve precise accumulation at lesion sites through functional modifications (e.g., magnetic targeting, ligand targeting), and enhance their ability to cross biological barriers (e.g., the blood-brain barrier). For example, co-loading corylin (a natural flavonoid) with chemotherapeutic agents into nanoparticles has been shown to significantly enhance antitumor efficacy (Zeng et al., 2023).

Future promise lies in closely integrating the development of intelligent nano-delivery systems with the zebrafish *in vivo* platform for rapid efficacy/toxicity validation. In zebrafish, the distribution of drug-loaded nanoparticles can be visualized and assessed in real time, along with their ability to accumulate at target sites (e.g., tumors or inflamed tissues), their efficiency in crossing the blood-brain or blood-retinal barriers, and their metabolic clearance processes (Persico et al., 2025). This integrated strategy of live visualization and efficacy evaluation for drug delivery can significantly accelerate the iterative optimization of novel delivery systems, ensuring their designs truly meet the demands of the complex *in vivo* environment. It thus provides an efficient screening and validation pathway to address the long-standing challenges of TCM ingredients being undeliverable and unsustainable at target sites (Knudsen et al., 2022).

### 6.4 Establishing standardized AI-assisted TCM diagnostic models

The standardization of syndrome differentiation remains a core bottleneck in the internationalization of TCM. Moving forward, zebrafish should be utilized to construct reproducible, quantifiable animal models of TCM syndrome with clearly associated biomarkers. By applying specific environmental, chemical, or genetic stressors (such as chronic stress to simulate “liver constraint”), and employing AI to perform cluster analysis of multidimensional phenotypes in zebrafish (including morphology, behavior, and physiology), stable and quantifiable animal phenotypic clusters corresponding to specific TCM patterns can be defined and linked to their unique molecular fingerprints (Guarin et al., 2021a). Simultaneously, by integrating AI-assisted digitization of tongue and pulse diagnostic information, a biological bridge can be established between these animal models and clinical TCM syndrome (Zhang et al., 2024c). This closed-loop approach — “digitization of clinical features - construction of syndrome models - verification through formula intervention” — provides a revolutionary standardized tool for research on disease-syndrome-formula correspondence.

### 6.5 Creating integrated systems pharmacology and AI-prediction platforms for TCM

Knowledge graphs integrating “disease - syndrome - target–formula” can be constructed to support clinical decision-making and novel drug discovery (Chen W. et al., 2025; Zheng et al., 2020). Furthermore, a “closed-loop AI phenomics” system can be developed. AI models trained on massive, multi-omics data from zebrafish can predict the therapeutic potential and toxicity risk of a new TCM formula based on its chemical composition input. This creates an intelligent “computational prediction - *in vivo* validation - feedback optimization” loop (LI T. et al., 2024), accelerating the discovery and optimization of safe and effective formulae, and fundamentally bridging TCM’s holistic perspective with modern systems pharmacology (Galkin et al., 2025).

Ultimately, it is essential to integrate all the aforementioned research directions to construct a comprehensive platform for systems pharmacology and AI prediction, centered on zebrafish as the core experimental engine. This platform will integrate a knowledge graph of “disease-pattern-target-formula” (Chen W. et al., 2025; Zheng et al., 2020). AI models will initially predict the multi-target interaction networks and potential phenotypes of TCM formulas based on their chemical compositions. Subsequently, these predictions will be validated through high-throughput, multidimensional systemic assays in live zebrafish, encompassing efficacy, toxicity, and multi-organ functional impacts. The massive volume of *in vivo* data generated from these validations will be fed back in real time to refine and train more accurate AI prediction models.

This intelligent closed loop will not only significantly accelerate the discovery and optimization of new, safe, and effective TCM formulas but also fundamentally bridge the gap between the holistic philosophy of TCM and modern systems biology (Galkin et al., 2025). It will establish zebrafish as a definitive integrator, dynamically elucidating and connecting the logic of TCM from the molecular level to the biological whole.

In summary, zebrafish is not intended to replace other technologies but serves as a dynamic, systemic *in vivo* integration platform. It links cutting-edge technologies—such as AI computing, multi-omics analysis, humanized models, nanotechnology, and phenomics—into an organic whole. Through this strategy, we can systematically address the core challenges in TCM research, ultimately advancing its modernization and internationalization. This approach will rejuvenate ancient wisdom in the language of modern biology and offer a unique Chinese solution for the prevention and treatment of complex diseases worldwide.

## 7 Conclusion

TCM has gained growing international recognition, yet its advancement is hindered by the absence of molecular biomarkers for TCM syndromes and the translational gap between model systems and clinical practice. The zebrafish model has emerged as a powerful tool for generating mechanistic hypotheses and performing early efficacy/safety profiling of TCM compounds across cardiovascular, neuropsychiatric, metabolic, and oncological disorders. For example, real-time imaging permits quantitative readouts such as dose-dependent bradycardia (e.g., percentage heart rate reduction), inhibition of immune cell migration distance, and reduction in tumor xenograft fluorescence, linking multi-target effects to measurable phenotypic endpoints.

Unlike well-defined small molecules or biologics, TCM research often contends with unidentified active ingredients, elusive multi-component synergies, and uneven quality control. As reviewed by Wang et al., many TCM formulas treat hyperuricemic nephropathy through dual-regulation of both the gut and kidneys (Wang T. et al., 2025), precisely the type of multi-organ pharmacological orchestration that zebrafish models are uniquely positioned to decipher. However, zebrafish differ from mammals in key physiological and metabolic aspects, and their larvae lack a fully mature adaptive immune system. Therefore, the model is best utilized for mechanistic exploration and early-stage pharmacological prioritization, not as a definitive predictor of clinical efficacy. All mechanistic insights and safety signals derived from zebrafish must be confirmed in mammalian models, with particular attention to long-term toxicity, reproductive toxicity, and adaptive immune responses that cannot be adequately captured in fish.

The proposed “zebrafish-plus” paradigms - integrating organoids, multi-omics, and AI-driven analytics - aim to strengthen the model’s translational value by enabling more quantitative, multi-scale pharmacological analyses. Rather than simply validating that TCM works in zebrafish, the goal is to use this platform to dissect specific, quantifiable multi-target mechanisms and generate robust hypotheses that can be tested in a tiered validation pipeline encompassing zebrafish, mammalian models, and human-relevant organoid or hiPSC systems. This cautious, evidence-based approach will be essential to unlocking the potential of TCM in the era of precision medicine.
